# Tunable high quality factor in two multimode plasmonic stubs waveguide

**DOI:** 10.1038/srep24446

**Published:** 2016-04-14

**Authors:** Zhiquan Chen, Hongjian Li, Shiping Zhan, Boxun Li, Zhihui He, Hui Xu, Mingfei Zheng

**Affiliations:** 1 College of Physics and Electronic, Central South University, Changsha 410083, China; 2 College of Communication and Electronic Engineering, Hunan City University, Yiyang 413000, China

## Abstract

We numerically investigate the optical characteristics of a metal-dielectric-metal (MDM) waveguide side-coupled with two identical multimode stub resonators. Double plasmon-induced transparency (PIT) peaks with narrow full width at half maximum (FWHM) and high quality factor (Q-factor) can be observed in this structure. The Q-factors of PIT peaks in two stub resonators system are larger than those in single stub resonator system. A multimode coupled-radiation oscillator theory (MC-ROT), which is derived from ROT, is proposed to analyze the spectral response in the multimode system for the first time. The analytical results are confirmed by the finite-difference time-domain (FDTD) simulation results. We can also find that the Q-factors of the two PIT peaks have an opposite evolution tendency with the change of the stubs parameters and the maximum can reach to 427. These results may provide some applications for ultrasensitive sensors, switches and efficient filters.

Surface Plasmon Polaritons (SPPs) have recently attracted great scientific interest because of their capability of controlling light in a sub-wavelength regime^1^. Among various plasmonic devices, the metal-dielectric-metal (MDM) waveguide, which supports the propagation of SPPs in the metal-dielectric interface and manipulates light on a sub-wavelength scale, can be regarded as an ideal integrated photonic device^2^,^3^. Due to the excellent features of easy fabrication and having deep sub-wavelength confinement of light with an acceptable propagation length for SPPs, MDM waveguide has been widely applied in Plasmon-induced transparency (PIT)^4^,^5^,^6^, optical filter^7^,^8^,^9^,^10^,^11^, switch^12^,^13^ and plasmonic sensor^14^,^15^,^16^,^17^,^18^.

Based on the unique features of MDM waveguide, PIT can be observed in coupled optical resonator systems, which were theoretically predicted and experimentally demonstrated in recent researches^19^,^20^,^21^,^22^,^23^,^24^,^25^,^26^,^27^. The stub resonator based on MDM waveguide has advantages of small size and simple fabrication technique. Chen *et al.* experimentally and numerically showed the plasmonic analogy of electromagnetically induced transparency (EIT) transmission in terahertz asymmetric waveguide with two stubs^20^. Huang *et al.* demonstrated the EIT-like in periodic-stub-assisted plasmonic waveguides^28^. Cao *et al.* used the Couple Mode Theory and the finite-difference time-domain (FDTD) to investigate the PIT in a bus waveguide coupled with two stub resonators^23^,^24^. Chen *et al.* numerically predicted a multiple PIT can be obtained in a MDM waveguide side-coupled with a series of stub resonators^29^. All of these are based on the single resonance mode in each stub resonator. Recently, Cao *et al.* used a single multimode stub resonator plasmonic system to realize double PIT^30^. However, due to the complex interaction between the multiple modes, the study about multiple multimode stub resonators system is rarely reported.

In this paper, we propose a MDM waveguide coupled with two identical multimode stub resonators. A double PIT spectrum with higher transmission and quality factors (Q-factors) than those of single multimode stub resonator system can be observed. We extend the radiation oscillator theory (ROT)^31^ and propose a multimode coupled-ROT (MC-ROT) to investigate the spectral response theoretically for the first time. The analytical results accord well with the numerical results, which provide an effective theoretical analysis method for multiple multimode stubs coupled to MDM waveguide. In addition, the Q-factor is mainly influenced by the structural parameters can reach to 427. The Q-factors of the two PIT peaks have an opposite evolution tendency with the change of stubs parameters. The proposed structure is easy to fabricate and this work may pave the way for the realization of highly integrated and effective optical devices, such as the ultrasensitive sensors, efficient switches and narrowband filters.

## Structure model and theoretical analysis

**Figure 1:**
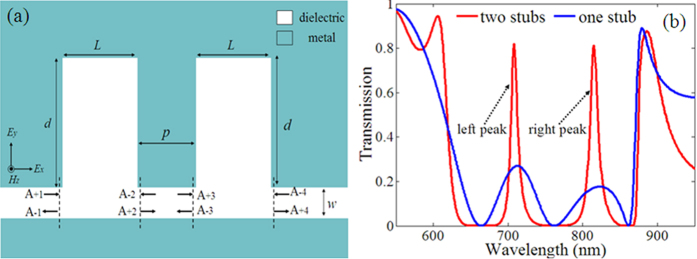
(**a**) Schematic of MDM waveguide side-coupled with two stub resonators. (**b**) Transmission spectra of MDM waveguide side-coupled with one stub (blue curve) and two stubs (red curve) when *L* = 400 nm, *d* = 600 nm.

Figure 1(a) schematically shows MDM waveguide side-coupled with two identical stub resonators. The dielectric and metal in the structure are air and silver, respectively. The main structure parameters are the width (*w* = 100 nm) of bus waveguide, length (*d*) and width (*L*) of the stub resonator, and the distance between the two stubs is *p* (*p* = 100 nm). A Gaussian light pulse with wide wavelength profile (from 600 nm to 1200 nm, Bandwidth = 250 nm) and normalized amplitude (Amplitude = 1) is incident along *x*-axis, SPP wave can be formed on metal-dielectric interface and confined in the waveguide. The width (*L*) of the stub resonators is large enough that the resonators can provide multiple modes.

When *d* = 600 nm, *L* = 400 nm, transmission spectra of a single and two multimode stubs coupled to bus waveguide are depicted in Fig. 1(b), respectively. The blue curve represents double PIT peaks can be observed in single multimode stub resonator system. The transmission is greatly enhanced and the PIT peaks become very sharp with narrow bandwidth when the bus waveguide coupled with two multimode stub resonators. The transmission of the left peak increases from 28% to 82%, and the full width at half maximum (FWHM) decreases from 50 nm to 10 nm. The transmission of the right peak increases from 18% to 80%, and the FWHM decreases from 63 nm to 13 nm. The Q-factor is defined as *Q* = *λ*/Δ*λ* (here*λ* is the wavelength of the peak and Δ*λ* is the FWHM of the PIT windows), which increases from 14 to 71 and 13 to 63 for the left peak and right peak, respectively.

**Figure 2:**
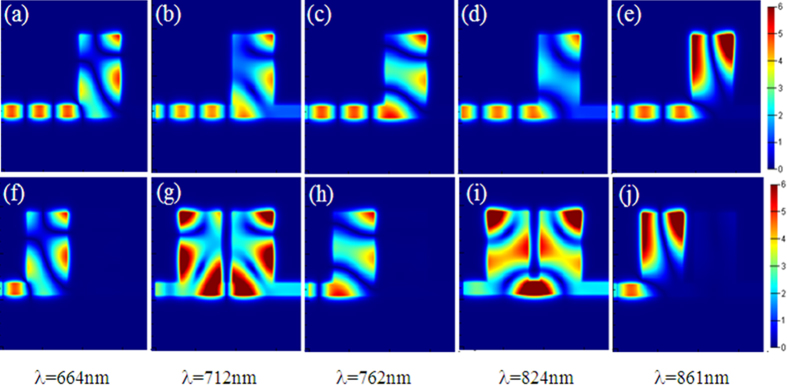
Magnetic field distributions of MDM waveguide side-coupled with single stub and two stubs for *L* = 400 nm and *d* = 600 nm at *λ* = 664 nm, 712 nm, 762 nm, 824 nm and 861 nm. (**a**–**e**) are the magnetic field distribution of structure with one stub; (**f**–**j**) are the magnetic field distribution of structure with two stubs.

In order to realize the physical mechanism of the changes in Fig. 1(b), we display the field distributions of the MDM waveguide coupled with a single stub and two stubs at *λ* = 664 nm, 712 nm, 762 nm, 824 nm, 861 nm in Fig. 2(a–j), respectively. Among these wavelengths, *λ* = 664 nm, 762 nm, 861 nm are the resonance wavelengths and *λ* = 712 nm, 824 nm are the PIT peaks wavelengths of stub with *d* = 600 nm, *L* = 400 nm. The resonance modes can be denoted as TM_*mn*_, where *m* and *n* denote the number of node of standing waves in horizontal and vertical directions in the stub resonator, respectively. There are three resonance modes simultaneously existing in each stub resonator, which can be expressed as TM_11_ (*λ* = 664 nm), TM_01_ (*λ* = 762 nm), and TM_10_ (*λ* = 861 nm), respectively. The two stub resonators and the connected waveguide act as a Fabry-Perot (FP) interferometer. The incident light can be reflected back and forward by each stub resonator. At the resonance wavelengths, the field distributions and intensity in the two stub systems are uniform with those in the single stub system. Most of the power is confined in the first stub or reflected back, with almost no power transport out, as illustrated in Fig. 2(a,c,e,f,h,j). At the PIT peaks wavelengths, a good part of the propagating radiation can exit the waveguide, and the rest can be reflected by the two stubs. There is a destructive interference between the two reflected waves in the waveguide, which reduces the reflected power and enhances the transmission, as depicted in Fig. 2(g,i). Moreover, the bandwidth of the transmission spectrum is inversely proportional to the effective length of the FP interferometer^32^. The effective length of the FP interferometer in two stub resonators system is larger than that in a single stub system, therefore, the PIT spectrum with narrow bandwidth can be observed in our proposed structure, as shown in Fig. 1(b).

According to ROT^31^, a single resonance mode motivated directly or indirectly by the input exists in each resonator. In our structure, there are three resonance modes directly motivated by the input in each stub resonator, simultaneously. Considering the mutual coupling among the three modes, we propose a MC-ROT to investigate the spectral response of the multimode system for the first time. The simple model with three coupled harmonic oscillators can be described as


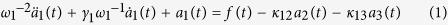



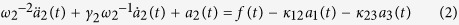



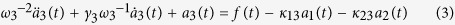


the three radiative oscillators with resonance frequency *ω*_*i*_ (*i* = 1, 2, 3) and damping factor *γ*_*i*_ (*i* = 1, 2, 3) are described by the excitation *a*_*i*_(*t*) (*i* = 1, 2, 3) and the external force *f*(*t*). Every two oscillators are linearly coupled with coupling strength *κ*_*ii*’_ (*i* ≠ *i*’). For simplicity, we set the coupling strength *κ*_12 =_*  κ*_23_ =*  κ*_13 =_*  κ*, approximately.

Equations (1)–(3), 2, 3 can be solved in the frequency domain by assuming a solution of the form *a*_*i*_(*t*) = *a*_*i*_(*ω*)·exp(−*iωt*) and *f*(*t*) = *f*(*ω*)·exp(−*iωt*), the electric current sheet with surface conductivity *σ*_*se* =_ ** *−iω* [*a*_1_(*ω*) + *a*_2_(*ω*) + *a*_3_(*ω*)]/*f*(*ω*) is introduced to describe this effective response, which can be written as


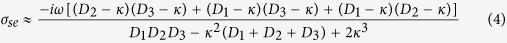


where *D*_*i*_ = 1−(*ω*/*ω*_*i*_)^2^−*iγ*_*i*_ (*ω*/*ω*_*i*_). So the transmission can be calculated in the following form^31^


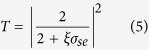


where *ξ* = ** *β*(*L*)*L/ωε*_*0*_*ε*_*i*_^33^ is the wave impedance, *ε*_*0*_ is the permittivity of vacuum, *ε*_*i*_ is the relative permittivity of the filled medium in resonators and *β*(*L*) is the propagation constant in MDM resonators. Using the transfer matrix method (TMM)^33^ and expanding the oscillator model to the second stub, the transmission of the two stubs coupled structure can be expressed as


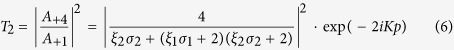


where *K* = ** *α*+i*β* is the Bloch wave vector. Using the theoretical analysis mentioned above, we can analyze the transmission spectra in the multiple multimode resonator systems.

## Simulation results and discussions

**Figure 3:**
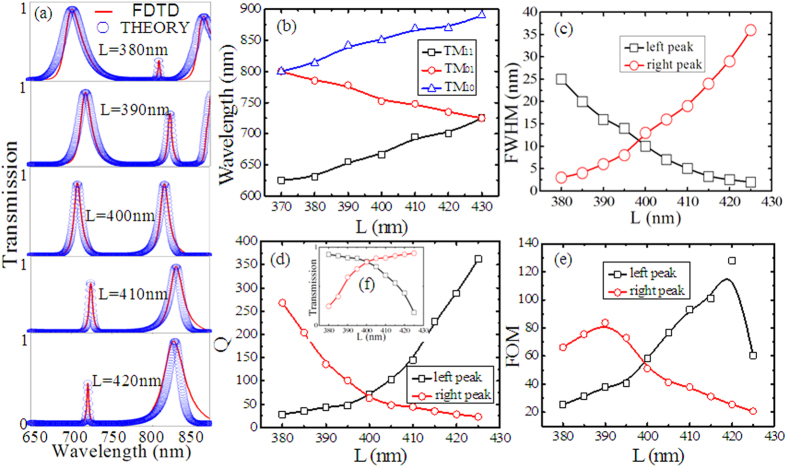
(**a**) Transmission spectra versus different *L*. The red solid curves are the simulation results and the blue circles are theoretical fittings. The other geometrical parameters are *w* = 100 nm, *d* = 600 nm, *p* = 100 nm. (**b**) The resonant wavelengths (*λ*_1_, *λ*_2_, *λ*_3_) of TM_11_, TM_01_ and TM_10_ modes versus *L*. (**c**–**f**) The FWHM, Q-factors, FOM and transmission corresponding to the two transparent peaks for different *L*, respectively. The black curve with squares represents the values of the left peak and the red curve with circles represents the values of the left peak. The fitting parameters are set as *κ* = 0.01; (*L* = 380 nm) [*λ*_1_, *λ*_2_, *λ*_3_] = [630, 785, 813] nm, [*γ*_1_, *γ*_2_, *γ*_3_] = [0.002, 0.001, 0.0013]; (*L* = 390 nm) [*λ*_1_, *λ*_2_, *λ*_3_] = [655, 778, 842]nm, [*γ*_1_, *γ*_2_, *γ*_3_] = [0.002, 0.001, 0.002]; (*L* = 400 nm) [*λ*_1_, *λ*_2_, *λ*_3_] = [666, 752, 850] nm, [*γ*_1_, *γ*_2_, *γ*_3_] = [0.0015, 0.001, 0.0015]; (*L* = 410 nm) [*λ*_1_, *λ*_2_, *λ*_3_] = [695, 748, 869] nm, [*γ*_1_, *γ*_2_, *γ*_3_] = [0.001, 0.001, 0.0012]; (*L* = 420 nm) [*λ*_1_, *λ*_2_, *λ*_3_] = [700, 735, 870] nm, [*γ*_1_, *γ*_2_, *γ*_3_] = [0.0008, 0.001, 0.0008].

We further investigate the spectral response of the proposed plasmonic waveguide system with different structural parameters. Figure 3 shows the transmission characteristics for the system with two identical stubs under different width *L*. Here, the other structural parameters are the same as those in Fig. 1(a).

As shown in Fig. 3(a,c), the transmission characteristics of the two peaks show different evolution trend as *L* increases. The left peak has a high transmission and broad bandwidth while the right peak possesses a low transmission and narrow bandwidth when *L* = 380 nm. As *L* increases, the transmission and the FWHM decrease for the left peak and increase for the right peak. The FDTD simulations are in excellent agreement with the theoretical fittings. Particularly, the right peak almost disappears when L = 370 nm and the left peak vanishes when *L* = 430 nm. This phenomenon can be attributed to the resonance wavelengths shift as *L* increases. As Fig. 3(b) shows, the resonance wavelengths of TM_11_ and TM_10_ have a red-shift while TM_01_ has a blue-shift with the increasing of *L*. The left and right peaks originate from the destructive interference between TM_01_ and TM_11_, TM_01_ and TM_10_, respectively. The resonance wavelengths of TM_01_ and TM_10_ overlap and there is about 150 nm wavelength detuning between TM_11_ and TM_01_ when *L* = 370 nm. Hence, the right peak almost disappears while the left peak has a high transmission and broad bandwidth in the transmission spectrum. With the increase of *L*, the wavelength detuning between TM_01_ and TM_10_ enlarges, resulting in an increase for the transmission and FWHM of the right peak. However, the wavelength detuning between TM_11_ and TM_01_ has an opposite trend, the transmission and FWHM of the left peak decrease as *L* increases.

The Q-factors of PIT peaks are also investigated and depicted in Fig. 3(d). It is seen that the changes of *L* also have different effects on the Q-factors of the two peaks. The Q-factor of the left peak increases from 28 to 362 as *L* ranges from 380 nm to 425 nm, while the Q-factor of the right peak decreases from 268 to 23. The inset Fig. 3(f) is the transmission of the two peaks as a function of *L*. When the Q-factor increases (decreases), the transmission gets weakened (enhanced), a tradeoff between Q-factor and transmission can be observed. In order to quantize the tradeoff, the product of Q-factor and transmission (*T*) is defined as the figure of merit (FOM = Q*T). The FOM of the two peaks with different *L* is plotted in Fig. 3(e), the maximum FOM = 128 for the left peak with a Q-factor of 288 and transmission of 44% when *L* = 420 nm, and the maximum FOM = 84 for the right peak with a Q-factor of 136 and transmission of 62% when *L* = 390 nm.

**Figure 4:**
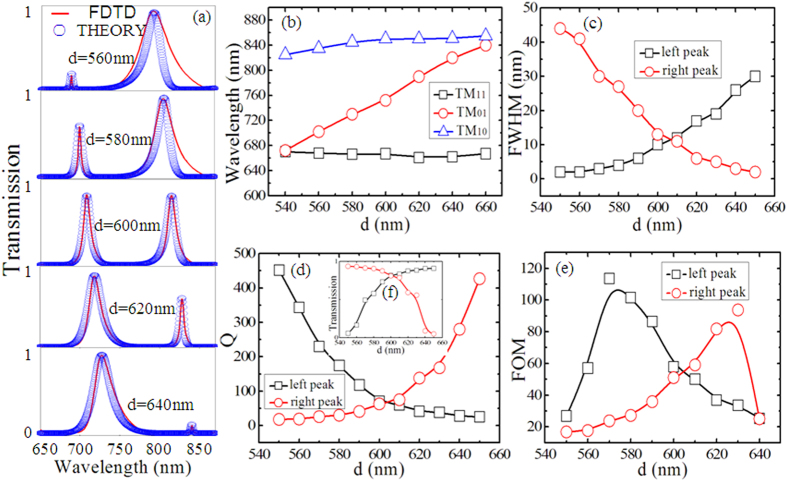
(**a**) Transmission spectra versus different *d*. The red solid curves are the simulation results and the blue circles are theoretical fittings. The other geometrical parameters are *w* = 100 nm, *L* = 400 nm, *p* = 100 nm. (**b**) The resonant wavelengths (*λ*_1_, *λ*_2_, *λ*_3_) of TM_11_, TM_01_ and TM_10_ modes versus *d*. (**c**–**f**) The FWHM, Q-factors, FOM and transmission corresponding to the two transparent peaks for different *d*, respectively. The black curve with squares represents the values of the left peak and the red curve with circles represents the values of the left peak. The fitting parameters are set as *κ* = 0.01; (*d* = 560 nm) [*λ*_1_, *λ*_2_, *λ*_3_] = [668, 702, 835] nm, [*γ*_1_, *γ*_2_, *γ*_3_] = [0.0045, 0.001, 0.0004]; (*d* = 580 nm) [*λ*_1_, *λ*_2_, *λ*_3_] = [666, 730, 845] nm, [*γ*_1_, *γ*_2_, *γ*_3_] = [0.003, 0.001, 0.0004]; (*d* = 600 nm) [*λ*_1_, *λ*_2_, *λ*_3_] = [666, 752, 850] nm, [*γ*_1_, *γ*_2_, *γ*_3_] = [0.0015, 0.001, 0.0015]; (*d* = 620 nm) [*λ*_1_, *λ*_2_, *λ*_3_] = [661, 790, 850] nm, [*γ*_1_, *γ*_2_, *γ*_3_] = [0.0015, 0.001, 0.0015]; (*d* = 640 nm) [*λ*_1_, *λ*_2_, *λ*_3_] = [662, 820, 851] nm, [*γ*_1_, *γ*_2_, *γ*_3_] = [0.001, 0.001, 0.003].

At last, we investigate the impact of the stub resonators length *d* on transmission characteristics in this plasmonic waveguide system. As shown in Fig. 4(a,c), the transmission and bandwidth increase for the left peak and decrease for the right peak as *d* increases. This evolution trend is opposite to those in Fig. 3. The FDTD simulation results are well consistent with the theoretical ones. The wavelengths of the three resonance modes versus *d* are plotted in Fig. 4(b). For various *d*, the resonance wavelengths of TM_11_ and TM_10_ are nearly constant. However, there is a liner relationship for the TM_01_ mode. The resonance wavelengths of TM_01_ and TM_11_ nearly overlap and the wavelength detuning of 200 nm is found between TM_01_ and TM_10_ when *d* = 530 nm. Therefore, the left peak almost disappears and the right peak has a high transmission and broad bandwidth in the spectrum. With the increase of *d*, the wavelength detuning between TM_01_ and TM_11_ enlarges, the transmission and FWHM of the left peak increase. However, the wavelength detuning between TM_10_ and TM_01_ has an opposite trend, the transmission and FWHM of the right peak decrease as *d* increases.

The Q-factors of the two peaks versus *d* are displayed in Fig. 4(d). The Q-factor of the left peak decreases from 339 to 25 as *d* increases, while the Q-factor of the right peak increases from 18 to 427. The inset Fig. 4(f) is the transmission of the two peaks as a function of *d*. It is worth noting that there is also a tradeoff between Q-factors and transmission. The FOM of the two peaks with different *d* is plotted in Fig. 4(e). The maximum FOM = 114 for the left peak with a Q-factor of 230 and transmission of 49% when *d* = 570 nm, and the maximum FOM = 94 for the right peak with a Q-factor of 168 and transmission of 56% when *d* = 630 nm. These results, obtained by fully considering the tradeoff, can provide some guidance for the design of efficient photonic devices.

## Conclusions

In summary, we have numerically and analytically demonstrated the PIT effect with high Q-factor in a MDM waveguide side-coupled with two multimode stub resonators. The Q-factors of PIT peaks in two stub resonators system are larger than those in the single stub resonator system. The coherence between the theoretical and numerical results validates the availability of the derived MC-ROT in effectively and conveniently describing the multimode system. By manipulating the parameters of the two stub resonators, a tunable Q-factor with a maximum of 427 can be obtained. In particular, the Q-factors of the two PIT peaks have an opposite evolution tendency with the change of the stubs parameters. With regard to the tradeoff between the Q-factor and the transmission, engineering the resonator geometry can lead to a maximum FOM of 128. Owning to the simple configuration and compactness, the high Q-factor structure has great potential applications in ultrasensitive sensors, optical switches and optical filters in integrated optical circuits.

## Methods

The frequency dependent optical property of the silver nanostructure is approximated by the Drude model: *ε*(*ω*) = *ε*_*∞*_ − *ω*_*p*_^2^*/*(*ω*^2^ + *iωγ*_*p*_), with *ω*_*p*_ = 1.38 × 10^16 ^s^−1^ is the bulk plasmon frequency, *ε*_*∞*_ = 3.7 and *γ*_*p*_ = 2.73 × 10^13 ^s^−1^ represents the damping rate. These values are obtained by fitting the experimental results report in^34^. With these parameters, the permittivity of sliver in Drude model agrees well with the experimental result in the visible and a part of near-infrared waveband, Drude model can be used to effectively simulate the optical properties of our structure in this waveband. The characteristic spectral responses of the structure are performed by the two-dimensional FDTD simulation. The spatial and temporal steps are set as Δ*x* = Δ*y* = 5 nm, and Δ*t* = Δ*x*/2c (c is the velocity of light in vacuum), respectively. We perform the FDTD simulations with a perfect matched layer (PML) boundary condition.

## Additional Information

**How to cite this article**: Chen, Z. *et al.* Tunable high quality factor in two multimode plasmonic stubs waveguide. *Sci. Rep.* **6**, 24446; doi: 10.1038/srep24446 (2016).

## References

[b1] Gramotnev D. & Bozhevolnyi S. Plasmonics beyond the diffraction limit. Nat. Photonics 4, 83 (2010).

[b2] Min C. & Veronis G. Absorption switches in metal-dielectric-metal plasmonic waveguides. Opt. Express 17, 10757 (2009). 19550473 10.1364/oe.17.010757

[b3] Huang Y., Min C. & Veronis G. Compact slit-based couplers for metal-dielectric-metal plasmonic waveguides. Opt. Express 20, 22233 (2012). 23037371 10.1364/OE.20.022233

[b4] He Z., Li H., Zhan S., Cao G. & Li B. Combined theoretical analysis for plasmon-induced transparency in waveguide systems. Opt. Lett. 39, 5543 (2014). 25360923 10.1364/OL.39.005543

[b5] Zhang Z., Zhang L., Li H. & Chen H. Plasmon induced transparency in a surface plasmon polariton waveguide with a comb line slot and rectangle cavity. Appl. Phys. Lett. 104, 231114 (2014).

[b6] Zhan S. *et al.* Theoretical analysis of plasmon-induced transparency in ring-resonators coupled channel drop filter systems. Plasmonics 9, 1431 (2014).

[b7] Zand I., Mahigir A., Pakizeh T. & Abrishamian M. Selective-mode optical nanofilters based on plasmonic complementary split-ring resonators. Opt. Express 20, 7516 (2012). 22453431 10.1364/OE.20.007516

[b8] Zhai X. *et al.* Tuning bandgap of a double-tooth-shaped MIM waveguide filter by control widths of the teeth. J. Opt. 15, 055008 (2013).

[b9] Wang G., Lu H., Liu X., Mao D. & Duan L. Tunable multi-channel wavelength demultiplexer based on MIM plasmonic nanodisk resonators at telecommunication regime. Opt. Express 19, 3513 (2011). 21369174 10.1364/OE.19.003513

[b10] Hu F., Yi H. & Zhou Z. Band-pass plasmonic slot filter with band selection and spectrally splitting capabilities. Opt. Express 19, 4848 (2011). 21445120 10.1364/OE.19.004848

[b11] Yun B., Hu G. & Cui Y. Resonant mode analysis of the nanoscal surface plasmo polariton waveguide filter with rectangle cavity. Plasmonics 8, 267 (2013).

[b12] Zhong Z., Xu Y., Lan S., Dai Q. & Wu L. Sharp and asymmetric transmission response in metal-dielectric-metal plasmonic waveguides containing Kerr nonlinear media. Opt. Express 18, 79 (2010). 20173825 10.1364/OE.18.000079

[b13] He Z. *et al.* Tunable Multi-switching in Plasmonic Waveguide with Kerr Nonlinear Resonator. Sci. Rep. 5, 15837 (2015). 26510949 10.1038/srep15837 PMC4625373

[b14] Lu H., Liu X., Mao D. & Wang G. Plasmonic nanosensor based on Fano resonance in waveguide-coupled resonators. Opt. Lett. 37, 3780 (2012). 23041857 10.1364/ol.37.003780

[b15] Chen J. *et al.* Coupled-resonator-induced Fano resonances for plasmonic sensing with ultra-high figure of merits. Plasmonics 8, 1627 (2013).

[b16] Chen Z., Cui L., Song X., Yu L. & Xiao J. High sensitivity plasmonic sensing based on Fano interference in a rectangular ring waveguide. Opt. Commun. 340, 1 (2015).

[b17] Chen Z. *et al.* Sensing characteristics based on Fano resonance in rectangular ring waveguide. Opt. Commun. 356, 373 (2015).

[b18] Zhan S. *et al.* Sensing analysis based on plasmon induced transparency in nanocavity-coupled waveguide. Opt. Express 23, 20313 (2015). 26367886 10.1364/OE.23.020313

[b19] Zhang Z., Zhang L., Li H. & Chen H. Plasmon induced transparency in a surface plasmon polariton waveguide with a comb line slot and rectangle cavity. Appl. Phys. Lett. 104, 231114 (2014).

[b20] Chen L. *et al.* Observation of electromagnetically induced transparency-like transmission in terahertz asymmetric waveguide-cavities systems Opt. Lett. 38, 1379 (2013). 23632490 10.1364/OL.38.001379

[b21] Han Z. & Bozhevolnyi S. Plasmon-induced transparency with detuned ultracompact Fabry-Perot resonators in integrated plasmonic devices. Opt. Express 19, 3251 (2011). 21369147 10.1364/OE.19.003251

[b22] Zhu Y., Hu X., Yang H. & Gong Q. On-chip plasmon-induced transparency based on plasmonic coupled nanocavities. Sci Rep 4, 3752 (2014). 24435059 10.1038/srep03752 PMC3894547

[b23] Cao G. *et al.* Formation and evolution mechanisms of plasmon-induced transparency in MDM waveguide with two stub resonators. Opt. Express 21, 9198 (2013). 23609630 10.1364/OE.21.009198

[b24] Cao G. *et al.* Uniform theoretical description of Plasmon-induced transparency in plasmonic stub waveguide. Opt. Lett. 39, 216 (2014). 24562110 10.1364/OL.39.000216

[b25] Piao X., Yu S. & Park N. Control of Fano asymmetry in plasmon induced transparency and its application to plasmonic waveguide modulator. Opt. Express 20, 18994 (2012). 23038539 10.1364/OE.20.018994

[b26] Lu H., Liu X., Mao D., Gong Y. & Wang G. Induced transparency in nanoscale plasmonic resonator systems. Opt. Lett. 36, 3233 (2011). 21847218 10.1364/OL.36.003233

[b27] Intaraprasonk V. & Fan S. Enhancing the waveguide-resonator optical force with an all-optical on-chip analog of electromagnetically induced transparency. Phys. Rev. A 86, 063833 (2012).

[b28] Huang Y., Min C. & Veronis G. Subwavelength slow-light waveguides based on a plasmonic analogue of electromagnetically induced transparency. Appl. Phys. Lett. 99, 143117 (2011).

[b29] Chen J., Wang C., Zhang R. & Xiao J. Multiple plasmon-induced transparencies in coupled-resonator systems. Opt. Lett. 37, 5133 (2012). 23258029 10.1364/OL.37.005133

[b30] Cao G. *et al.* Plasmon-induced transparency in a single multimode stub resonator. Opt. Express 22, 25215 (2014). 25401555 10.1364/OE.22.025215

[b31] Tassin P. *et al.* Electromagnetically induced transparency and absorption in metamaterials: The radiating two-oscillator model and its experimental confirmation. Phys. Rev. Lett. 109, 187401 (2012). 23215325 10.1103/PhysRevLett.109.187401

[b32] Svelto O. Principles of laser (Fifth edition). New York, Springer (2010).

[b33] Pannipitiya A., Rukhlenko I., Premaratne M., Hattori H. & Agrawal G. Improved transmission model for metal-dielectric-metal plasmonic waveguides with stub structure. Opt. Express 18, 6191 (2010). 20389642 10.1364/OE.18.006191

[b34] Palik E. Handbook of optical constants of solids. Boston, Academic Press (1985).

